# Improving Gender Equity for Women in Nephrology: A Global Perspective

**DOI:** 10.1016/j.ekir.2024.01.036

**Published:** 2024-01-24

**Authors:** Sabine Karam, Urmila Anandh, Aldjia Hocine, Michelle N. Rheault, Benedicte Sautenet, Maryvonne Hourmant

**Affiliations:** 1 Division of Nephrology and Hypertension, Department of Medicine, University of Minnesota, Minneapolis, Minnesota, USA; 2 Division of Nephrology and Hypertension, Department of Internal Medicine, American University of Beirut, Beirut, Lebanon; 3 Department of Nephrology, Amrita Hospitals, Faridabad, Delhi, National Capital Region, India; 4 Service de Néphrologie, Clinique du Landy, Saint-Ouen, France; 5 Service de Néphrologie, Hôpital Bichat Claude Bernard, AP-HP, Paris, France; 6 Division of Pediatric Nephrology, University of Minnesota Medical School, Minneapolis, Minnesota, USA; 7 Service de Néphrologie et Immunologie Clinique, CHRU de Tours, Tours, France; 8 Faculté de Médecine, Université Francois Rabelais, Tours, France; 9 Service de Néphrologie et Immunologie Clinique, University Hospital of Nantes, Nantes, France

### Introduction

Although the number of women pursuing a career in nephrology has been increasing worldwide, there remain barriers to career achievement. Today, thanks to women pioneers such as Drs Renee Habib, Marie-Claire Gubler, Sharon Anderson, Priscilla Kincaid-Smith, Saraladevi Naicker, and many others, gender is not a barrier to complete medical studies and elect nephrology as a specialty. In 2009, women represented just 33% of the nephrologists practicing in France; 14 years later, this number has increased to approximately 48%.^1^ In the United States, approximately 25% of nephrologists are women and 36% of fellows are women, suggesting that numbers will continue to improve over time.^2^ However, gender equity remains elusive when it comes to fulfilling career goals for those who enter the field. The glass ceiling is not breaking yet, and women remain underrepresented in leadership positions, academic medicine, and at medical meetings worldwide.^3^^,^^4^ In the United States, just 6% of department chairs are women and just 9% of division chiefs are women.^5^ The fate of women physicians in one of the oldest and largest countries of the world is no different. In ancient India, medical practice was the domain of priests. Because this was a male-dominated profession, absence of the mention of female medical practitioners was the rule. This attitude did not change over the centuries and women medical professionals in India had to struggle to gain acceptance. The first woman nephrologist, Professor V.N. Acharya not only chose against all odds nephrology as a career but went on to become one of the pioneers of the discipline by establishing one of the earliest nephrology programs in India. This editorial will illustrate the perspective of members of several scientific societies worldwide, notably the International Society of Nephrology, the French-speaking society of Nephrology (Société Francophone de Néphrologie, Dialyse et Transplantation), the Club des Jeunes Néphrologues in France, the Women in Nephrology (WIN) organization, and WIN-India, and highlight growing efforts worldwide that are trying to constructively address the issue of gender equity in nephrology.

### Challenges Faced by Women in Nephrology

Women in nephrology face many challenges, such as limited mentorship opportunities, gender bias when it comes to recognition and promotion, imposter syndrome, and the need for better work-life integration. A few of these issues have been confirmed in recent surveys distributed in the nephrology community. In a questionnaire sponsored and distributed by the Société Francophone de Néphrologie, Dialyse et Transplantation to the French speaking nephrology community with the help of Club des Jeunes Néphrologues and presented as an oral communication at the 2022 European Renal Association Congress,^6^ some of the identified obstacles to career advancement were “the lack of incentives by the civil society for women professional advancement” and “preference for a male professional whenever applying for a leadership position with equal qualifications.” In addition, “the absence of subsidized daycares,” “the lack of motivation to seek for a leadership position from women themselves,” and “the absence of or the insufficient duration of paternity leave” were also deemed in this survey to jeopardize career goals fulfillment for women. A web-based survey of its members by the Australian and New Zealand Society of Nephrology recorded the degree of perceived inequity on a Likert scale, ranging from 1 (none) to 5 (complete).^7^ Among the 134 respondents, of whom 57% were women, 88% perceived inequities in the workforce and 75% felt that gender was one of its drivers. Qualitative analysis highlighted limited opportunities for advancement and lack of formal assistance for those experiencing inequities. Respondents called for policy changes to ensure diverse representation on committees and in executive leadership positions. Nonetheless, the most important issue to solve for women nephrologists is likely motherhood and the difficulties in balancing the responsibilities of parenting and professional career. Globally, there are big discrepancies in access to parental leave and remuneration, and worldwide maternity leave varies between 0 and 40 weeks for physicians.^8^ In the United States, the Family and Medical Leave Act allows for 12 weeks of maternity leave, but this is unpaid.^9^ A lack of systems-level guidelines for pregnancy and parental leave, inconsistent workplace accommodations for pregnant and lactating physicians, a lack of guidance and support for planning parental leaves, and difficulties obtaining clinical coverage for parental leave could also play a significant role.^6^ Women physicians may delay childbearing, be at increased risk of adverse peripartum complications when they do become pregnant, and face subsequent discrimination and lower earnings. In that regard, 60% of the respondents to the Société Francophone de Néphrologie, Dialyse et Transplantation survey deemed it necessary to establish an adequate support system at work such as daycares and lactation rooms.^6^

### Overview of the Different Initiatives Geared at Achieving Gender Equity in Nephrology

Several initiatives have been established independently worldwide with the goal of developing mentorship and career development opportunities for women in the field. The oldest initiative is undoubtedly WIN, an independent nonprofit organization founded in 1983 by Drs. Nancy Gary, Lois Katz, Sandra Levison, and Mabel Purkerson in the United States. For years, WIN provided a space for networking and community for women at the annual American Society of Nephrology conference as well as hosted a premeeting professional development course through 2014. Currently, WIN hosts a yearly professional development conference for nephrologists to provide leadership training particularly for early-career women. In addition, WIN nominates women for awards in nephrology, provides a speaker’s bureau to allow for conference organizers to easily find a diverse list of speakers or panelists for any event, provides individualized mentoring for nephrologists and trainees, and advocates for family-friendly institutional and conference policies. WIN also provides a national leadership platform for its Executive Council members who have often moved on to other leadership positions in nephrology after their tenure with WIN. Finally, WIN promotes and shares content about achievements of women in nephrology via its social media channels to highlight their work and show women trainees the multiple career paths in nephrology that are available to them. Although there has been recent movement toward improved gender balance of the executive council of the American Society of Nephrology and other national nephrology organizations, women remain underrepresented in research, both grant funding and publications, and in academic leadership. The WIN movement has blossomed worldwide as its Indian counterpart was started in August 2021 with positive backing from the original WIN, the International Society of Nephrology and prominent global women leaders such as Professors Liz Lightstone, Valerie Luyckx, and Lisa Curtis.S1 The organization has since then conducted webinars, quizzes, symposiums, case discussions, and sent out newsletters. The newly formed Indian society also supported their counterparts in Africa to establish the “Women in Nephrology-Africa” movement, which took wings at the 2023 African Association of Nephrology meeting in Cairo. Within the Australian and New Zealand Society of Nephrology, a gender equity working group was set up in 2017, and in 2020, the Equity, Diversity, and Inclusivity committee was formed within the same society to address intersectional bias and barriers to diverse workforce representation for women and other minority groups.^7^ In addition, subsequent to the Société Francophone de Néphrologie, Dialyse et Transplantation survey and after having noticed that women were poorly represented in the leading committees of the society, women nephrologists from France and other French-speaking countries created in 2022 a commission called FEMKY (for “FEMmes” i.e., women and KY for kidney). The group adopted a plan of action with 10 objectives (Table 1), related to their professional and personal life, highlighting the need for solidarity, mentorship, and education. An important goal for FEMKY is to set up a support system for women exposed to abuse or harassment at work, a vital issue identified by this group from a recent survey.S2 Most recently, the 60th congress of the European Renal Association saw the birth of a European advocacy group for WIN. Finally, another notable initiative is the occurrence in 2017 of a workshop by the Women in Transplantation Committee, a subcommittee of The Transplantation Society of Australia and New Zealand that instituted several tangible changes.S3 They include the reporting on an annual basis of the gender split of invited speakers and session chairs at the annual scientific meeting to the council of the society with the aim of gender equality and the establishment of a subsidized child-minding facility. The growth of all these initiatives and their work augur well for the equitable growth of nephrology worldwide.

**Table 1:** The 10 objectives of the FEMKY group

1. Creation of a dedicated committee within the SFNDT (FEMKY)
2. Increase the visibility and promotion of women through parity in commissions, congress invitations as speakers and moderators
3. Create a network of women mentors
4. Highlight female role models
5. Organize self-confidence workshops for women nephrologists
6. Information on daily sexist behaviors within the nephrology community, including trainees
7. Introduce a gender equity chapter as part of the training curriculum of young nephrologists
8. Set up a watch initiative over gender disparity
9. Set up a help cell for women exposed to abuse or harassment at work
10. Promote actions regarding private life (pregnancies, care of young children) that could help promotion of women and improve life-work balance

FEMKY, “FEMmes” that is, women and KY for kidney; SFNDT, Société Francophone de Néphrologie, Dialyse et Transplantation.

### Conclusion

There remain significant disparities when it comes to women representation in leadership positions worldwide. It matters that the nephrology community determines the precise factors and finds pragmatic solutions to a global problem. Some of the fruitful initiatives achieved so far have been the establishment of organizations dedicated to the promotion and recognition of women nephrologists through tailored mentorship programs and advocacy for equitable representation in councils and executive committees of scientific societies along with equal opportunities to present at national and international conferences. Nonetheless, major gaps persist when it comes to achieving a smooth transition to motherhood and integrating this major life-changing event in a woman’s career without jeopardizing her aspirations. Improving and alleviating the struggles that many women go through to achieve their career goals will benefit all nephrologists through a more diverse, representative, efficient, and effective workforce leading to more scientific innovation. Ensuring that every trainee who chooses nephrology will be able to achieve her own path should be an everyday goal for our community.

## Disclosure

All the authors declared no conflicting interests.

## Supplementary Material

Supplementary File (PDF)

**Supplemental References.**
