# Supplementary material for: Improving Gender Equity for Women in Nephrology: A Global Perspective
Source: Kidney Int Rep. 2024 Jan 24;9(3):501–3. doi: 10.1016/j.ekir.2024.01.036 (PMC10927478; doi:10.1016/j.ekir.2024.01.036)
Supplement: Supplementary File (PDF) [file mmc1.pdf]

Supplementary References:

S1. Meena P, Parikh N, Mohan K, Bajpai D, Anandh U. Women in Nephrology-India: One-Year-old, Yet Miles to Cover. *Kidney International Reports*. 2023;8(3):688-689. doi:10.1016/j.ekir.2022.12.012

S2. Sautenet B, Karam S, Vrigneaud L, et al. SA-PO067.Assessment of Equity, Inclusiveness, and Quality of Life for French-Speaking Male and Female Nephrologists: A Survey by the FEMKY Group. J Am Soc Nephrol 34: 2023: 741.

S3. Dwyer KM, Clark CJ, MacDonald K, et al. Gender Equity in Transplantation: A Report from the Women in Transplantation Workshop of The Transplantation Society of Australia and New Zealand. *Transplantation*. 2017;101(10):2266-2270. doi:10.1097/TP.0000000000001900
